# Review of the referral documents of patients with malignant soft tissue tumors

**DOI:** 10.1038/s41598-022-24158-w

**Published:** 2022-11-14

**Authors:** Manabu Hoshi, Naoto Oebisu, Tadashi Iwai, Akiyoshi Shimatani, Yoshitaka Ban, Naoki Takada, Hana Yao, Hiroaki Nakamura

**Affiliations:** Department of Orthopedic Surgery, Osaka Metropolitan University Graduate School of Medicine, 1-4-3 Asahi-Machi, Abeno-Ku, Osaka, 545-8585 Japan

**Keywords:** Cancer, Oncology

## Abstract

Fifteen years have passed since the soft tissue tumor practice guidelines were first published in Japan. Tumor size of ≥ 5 cm and tumor depth were key findings suggestive of malignant soft tissue tumors. We reviewed the referral documents provided by the referring physicians to see if these two findings were reported. The study was conducted from January 2007 to December 2021 and included 142 patients (83 men and 59 women; median age, 64 [6–94] years) with malignant soft tissue tumors. Patient referral documents from physicians were screened for descriptions of the tumor size and depth. The tumor size, depth, and both were described in 51.4%, 36.6%, and 23.2% of the referrals, respectively. Both findings were mentioned in 23.8%, 21.7%, and 25.0% of referrals in 2007–2011, 2012–2016, and 2017–2021, respectively. Of orthopedic surgeons and other physicians, 61.2% and 38.6%, respectively, described the tumor size. Whether the general physicians could follow the soft tissue tumor practice guidelines was difficult to conclude by reviewing patient referral documents. However, orthopedic surgeons seemed to pay more attention to tumor size. Awareness regarding soft tissue tumor practice guidelines should be increased to help diagnose malignant soft tissue tumors early.

## Introduction

Malignant soft tissue tumors are very rare. Unplanned surgical treatments used to treat soft tissue tumors without considering the possibility of the malignant nature of the tumor can have serious consequences, such as local recurrences and skip metastases. These may result in devastating outcomes such as amputations^1^. When general practitioners encounter malignant soft tissue tumors in daily medical care, it is recommended that they promptly refer these patients to orthopedic oncologists^2,3^.

More than 15 years have passed since the Japanese Orthopaedic Association (JOA) clinical practice guidelines on the management of soft tissue tumors were first published in 2005^4^. They were revised in 2012^5^ and 2020^6^. According to these guidelines, a malignant soft tissue tumor should be suspected if the tumor size is over 5 cm and/or the tumor is deeply seated. Therefore, orthopedic oncologists usually assume that this clinical information is assessed by general physicians at the time of diagnosis. However, the extent of application of the clinical practice guidelines is unclear. In this study, we reviewed whether these two important clinical details were described in the patient referral documents by referring physicians and assessed whether the clinical practice guidelines conformed to daily medical care.

## Materials and methods

This study was designed as a retrospective analysis at a single institution. The following patients were included: (1) those with a pathologically diagnosed malignant soft tissue tumor, (2) those whose referral document from the previous physician could be analyzed, (3) those whose tumor size on magnetic resonance imaging (MRI) could be obtained at the initial visit, (4) those whose tumors were deeply seated, and (5) those with tumor size ≥ 5 cm.

In total, 243 patients diagnosed with malignant soft tissue tumors at our institution between January 2007 and December 2021 were selected from our institutional database. Among them, 101 patients were excluded because of incomplete medical information (23 patients), superficially seated tumors (57 patients), and tumor size of less than 5 cm (21 patients). The remaining 142 patients with typical findings of malignant soft tissue tumors were included in the study.

At their initial visit to our institution, the patients were evaluated radiologically using plain radiographs and MRI for the target lesions, and then the tumor size was measured. The presence of lung metastases was checked using computed tomography. Among 142 patients, needle biopsy was performed in 91 patients, and open biopsy was performed in 39 patients at our institution. The initial diagnosis was determined for the remaining 12 patients at their previous referring institutions. Incomplete resection was performed in 11 of the 12 patients, and malignant soft tissue tumors were pathologically suspected. The patients with a tumor sized ≥ 5 cm and at a deep location on MRI after incomplete resection were included in this study. One patient underwent open biopsy at another sarcoma center and was referred to our institution for treatment. All biopsy and surgically resected specimens were examined or reviewed by a pathologist specializing in sarcoma pathology; the diagnosis was based on the standard criteria for bone and soft tissue sarcoma subtyping^7^. All soft tissue sarcomas were graded according to the Fédération Nationale des Centres de Lutte Contre le Cancer system^8^, with grade 2 and 3 tumors considered high-grade. The clinical stage of each patient was evaluated according to the guidelines of the American Joint Committee on Cancer for soft tissue tumors^9^.

All patients with malignant soft tissue tumors were referred to our hospital by their previous doctors. Clinical information such as age at diagnosis, sex, involved site (forearm, upper limb, trunk, thigh, and lower leg), tumor stage, tumor size, and pathological diagnosis was obtained from patients’ medical charts and radiological investigations.

From the patient referral documents obtained from their referring physicians, details such as the presence or absence of the description of the “tumor size” and “tumor depth,” the specialty of each previous physician, and the affiliated institution (local hospital or clinic) were obtained. For the description of the tumor size, comparative expressions describing tumor size of over 5 cm as “head-sized” or “basketball-sized” were also acceptable, in addition to the actual measured size.

The diagnosis period was divided into three 5-year groups as follows: Group 1, 2007–2011; Group 2, 2012–2016; and Group 3, 2017–2021. The frequencies of descriptions of “tumor size” and “tumor depth,” endorsed in the referral documents were also compared based on the previous physician’s specialty (orthopedic surgeon or non-orthopedic physicians) and the affiliated institution of the previous physician (local hospital or clinic).

This study was performed in accordance with relevant guidelines/regulations, and was approved by the institutional review board of the Osaka Metropolitan University Graduate School of Medicine (approval number: 4394). This study was a retrospective chart review; thus, consent for participation was waived and approval of this waiver was obtained by the institutional review boards at Osaka Metropolitan University Hospital.

### Statistical analysis

The Mann–Whitney U test and Fisher’s exact probability test were performed to statistically compare the two groups. Statistical significance was set at* p* < 0.05. Statistical analysis was performed using the Excel Statistics software for Windows (version 2022; SSRI Co., Ltd., Tokyo, Japan).

### Consent to participate

Since this study was a retrospective chart review, consent for participation was waived and approval of this waiver was obtained by the institutional review boards at Osaka Metropolitan University Hospital.

## Results

The clinical information of the 142 patients is shown in Table 1. The patients comprised 83 men and 59 women; the median age at diagnosis was 64 years (range 6–94 years). All tumor sizes were over 5.0 cm and the median tumor size was 11.0 cm (5.0–28.3 cm). TNM staging of the tumors was stage IB in 40 cases, IIIA in 35 cases, IIIB in 48 cases, and IV in 19 cases. The affected sites were the upper arms and thighs in 82 cases, trunk in 26, lower legs in 14, upper arms in 13, and forearms in seven cases.

**Table 1:** Demographic data of participants.

Factors	Number	
**Age**		
Years (Mean)	64	6–94
**Sex**		
Male	83	58.5%
Female	59	41.5%
**Tumor size**		
cm	11.0	5.0–28.3
**TNM staging**		
IB	40	28.2%
IIIA	35	24.6%
IIIB	48	33.8%
IV	19	13.4%
**Affiliated Institution of previous doctors**		
Hospital	100	70.4%
Clinic	42	29.6%
**Specialty of previous doctors**		
Orthopedic surgeon	98	69.0%
General surgeons	18	12.7%
Internal physician	11	7.7%
Plastic surgeon	6	4.2%
Dermatologist	3	2.1%
Anesthesiologist	2	1.4%
Brain surgeon	1	0.7%
Otorhinolaryngologist	1	0.7%
Obstetrician and gynecologist	1	0.7%
Pediatrician	1	0.7%
**Affected site**		
Thigh	82	57.7%
Lower leg	14	9.9%
Upper arm	13	9.2%
Forearm	7	4.9%
Trunk	26	18.3%
**Diagnosis year**		
Group 1 (2007–2011)	42	29.6%
Group 2 (2012–2016)	60	42.3%
Group 3 (2017–2021)	40	28.1%

The affiliated institutions of the referring physicians were hospitals in 100 cases (70.4%) and clinics in 42 cases (29.6%). The referring physicians were as follows: orthopedic surgeons in 98 cases (69.0%), general surgeons in 18 cases (12.7%), internal physicians in 11 cases (7.8%), plastic surgeons in six cases (4.2%), dermatologists in three cases (2.1%), anesthesiologists in two cases (1.4%), brain surgeon in one case (0.7%), otorhinolaryngologist in one case (0.7%), obstetrician and gynecologist in one case (0.7%), and pediatrician in one case (0.7%). The number of patients referred to our institution was 42 (29.6%) in 2007–2011 (Group 1), 60 in 2012–2016 (Group 2), and 40 in 2017–2021 (Group 3).

The histopathological features of the different tumors are presented in Table 2.

**Table 2:** Histopathological features of the tumors.

Histopathology	Number
**Liposarcoma**	
Well differentiated	31
Myxoid	20
Pleomorphic	13
Dedifferentiated	2
Undifferentiated pleomorphic sarcoma	20
Myxofibrosarcoma	12
Rhabdomyosarcoma	8
Leiomyosarcoma	6
Malignant peripheral nerve sheat tumor	6
Low grade fibromyxoid sarcoma	5
Solitary fibrous tumor	4
Epithelioid sarcoma	3
Synovial sarcoma	3
Extraskeletal myxoid chondrosarcoma	3
Extraskeletal Ewing sarcoma	2
Alveolar soft part sarcoma	1
Pecoma	1
Fibrosarcoma	1
Extraskeletal osteosarcoma	1

**Figure 1:**
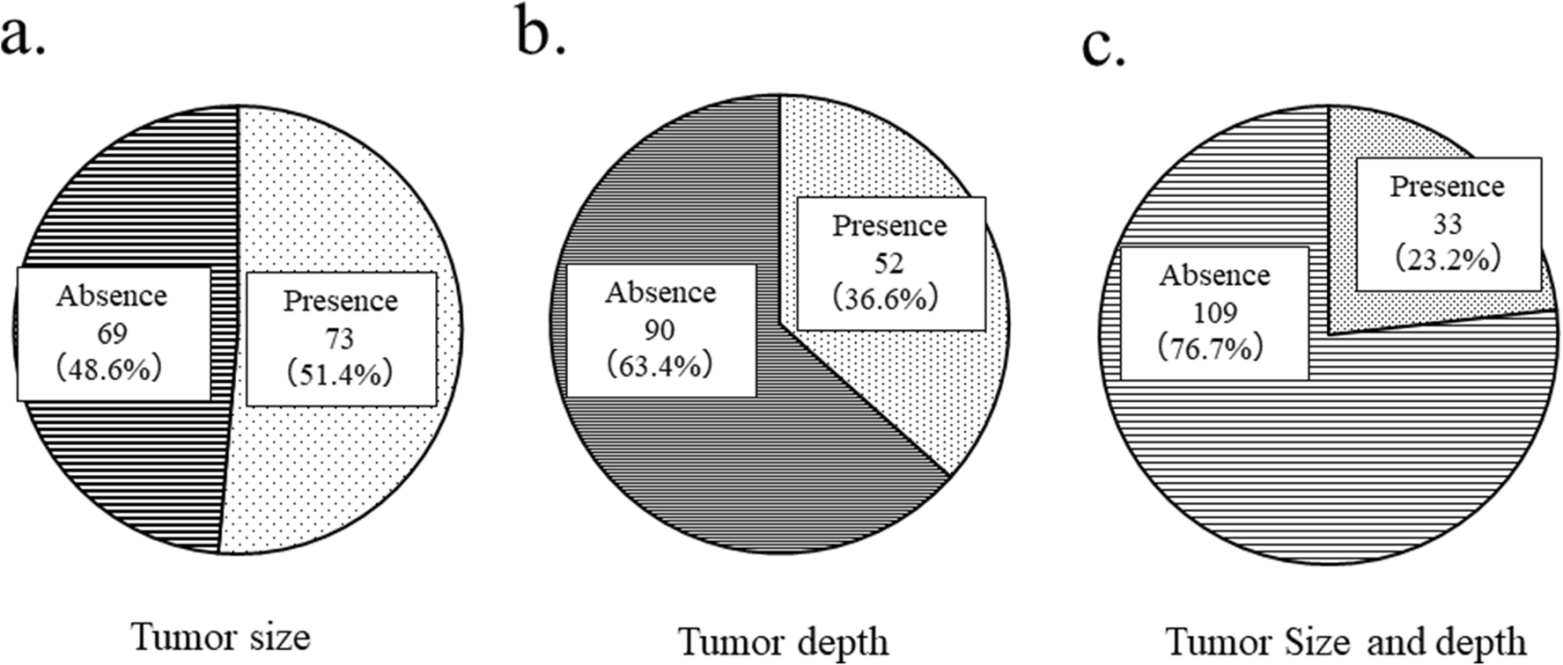
Description of tumor information in the patient referral document. (**a**) Tumor size was described in 73 cases (51.4%). (**b**) Tumor depth was described in 52 cases (36.6%). (**c**) Both tumor depth and size were described in 33 cases (23.2%).

After examining 142 patient referral documents of malignant soft tissue tumors, we found that the tumor size (Fig. 1a) was described in 73 cases (51.4%) and not described in 69 cases (48.6%). The tumor depth (Fig. 1b) was described in 52 cases (36.6%) and not described in 90 cases (63.4%). Both tumor size and location (Fig. 1c) were described in only 33 cases (23.2%).

**Figure 2:**
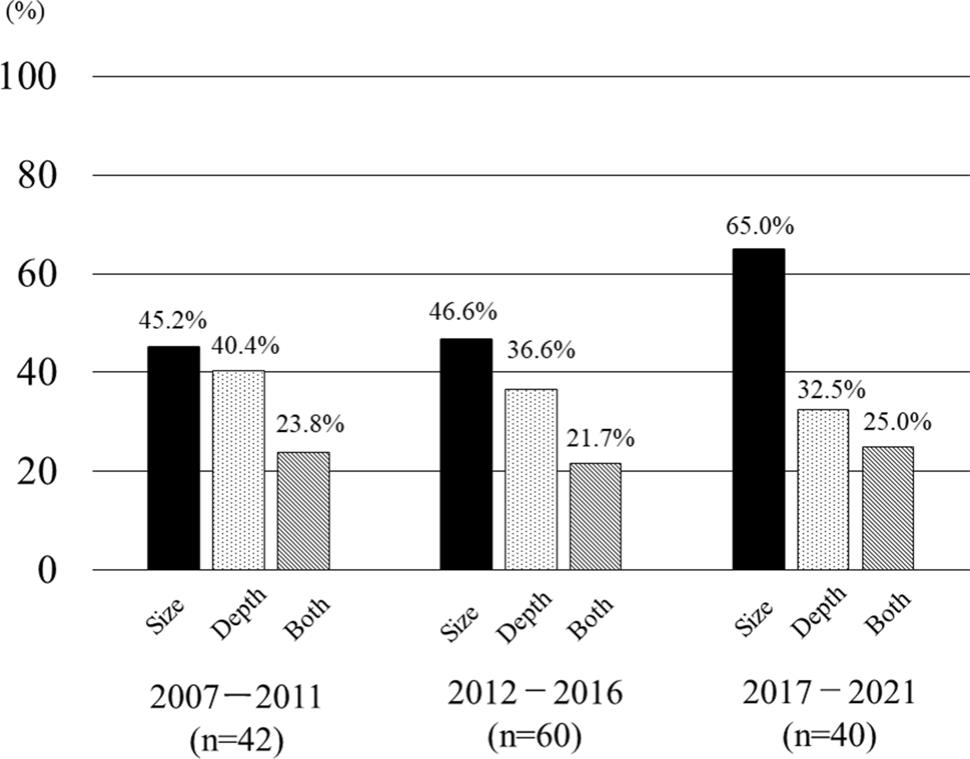
Description of tumor information in patient referral document every 5 years.

The description of the tumor size was present in 45.2%, 46.6%, and 65.0% of the patient referral documents of Group 1 (2007–2011: n = 42), Group 2 (2012–2016: n = 60), and Group 3 (2017–2022: n = 40), respectively (Fig. 2), indicating a gradual improvement in the reporting. The tumor depth was described in the referral documents of Groups 1, 2, and 3 (40.4%, 36.6%, and 32.5%, respectively), showing a persistently low rate. The description rate of both the tumor size and depth in Groups 1, 2, and 3 was 23.8%, 21.7%, and 25.0%, respectively.

The affiliated institutions of the previous physicians were divided into local hospitals and clinics (Table 3). Of 101 referring physicians working in hospitals, 53 (52.4%) described the tumor size, 41 (40.6%) described the tumor depth, and 25 (24.8%) described both the tumor size and depth. Of 41 referring physicians working in their own clinics, 24 (41.2%) described the tumor size, 11 (26.9%) described the tumor depth, and eight (19.5%) described both the tumor size and depth. There were no statistically significant differences between them.

**Table 3:** Comparisons between the affiliated institutions of referring physicians.

	Hospital	Clinic	*p* Value
	(N = 101)	(N = 41)	
Tumor size	53	24	0.51
Location	41	11	0.12
Both	25	8	0.66

The referring physicians were divided into orthopedic surgeons and non-orthopedic physicians (Table 4). Of 98 orthopedic surgeons, 60 (61.2%) described tumor size, 38 (38.8%) described tumor depth, and only 26 (26.5%) described both the tumor size and location. Of 44 non-orthopedic physicians, 17 (38.6%) described the tumor size, 14 (31.8%) described the tumor size, and only seven (15.9%) described both the tumor size and location. There was a statistically significant difference between them (*p* = 0.01) with regard to the description of the tumor size, suggesting that orthopedic surgeons are more aware of the importance of reporting the tumor size than physicians who are not orthopedicians.

**Table 4:** Comparisons between orthopedic surgeons and non-orthopedic physicians.

	Orthopedic surgeon	Non-orthopedic surgeon	*p* Value
	(N = 98)	(N = 44)	
Tumor size	60	17	0.01
Location	38	14	0.43
Both	26	7	0.27

## Discussion

Malignant soft tissue tumors are very rare. However, benign soft tissue tumors, such as lipoma, schwannoma, and hemangioma, are relatively common^10^. The incidence rate of benign soft tissue tumors was estimated to be approximately more than 100 times that of malignant tumors^4–6^. Therefore, some general surgeons, without suspecting a malignancy, remove the soft tissue tumor. Generally, this does not lead to any complications because of the high incidence of benign soft tissue tumors^4–6^. However, if the resected tumor is pathologically diagnosed as malignant, there may be serious consequences. This inappropriate management begins with a misdiagnosis and a disregard for or violation of the recommendations of the guidelines.

In Japan, as of March 2022, the number of registered qualified orthopedic oncologists is only 199 (0.8%) out of the total 25,769 orthopedic surgeons. The management of malignant soft tissue tumors requires sufficient knowledge, special training, multidisciplinary teams, and even experience. Currently, to prevent the inappropriate management of malignant soft tissue tumors, patients with malignant soft tissue tumors are recommended to be referred to specialized institutions, such as local cancer center institutions and university hospitals^11^.

More than 15 years have passed since the first version of the JOA clinical practice guideline was published. Regarding the diagnosis of malignant soft tissue tumors, the guidelines mentioned that a tumor “size ≥ 5 cm” and “deep location” are typical findings suggestive of malignant soft tissue tumors, especially among orthopedic oncologists. Nevertheless, in practice, the inappropriate management of malignant soft tissue sarcomas has been continuously reported^12–14^. Therefore, it is necessary to obtain feedback regarding physicians’ commitment to this guideline.

The result of this study showed that the tumor size was described in 51.4% of patient referral documents provided by patients’ referring physicians, and tumor depth was described in 36.6%. Only 23.3% of referring physicians described both the findings. The description rate of the tumor size increased to 65.0% in the previous 5 years, but there was no change in the reporting of the tumor depth, indicating that physicians are paying more attention to the tumor size during examination (Fig. 2).

In this study, 98 (69.0%) of referring physicians were orthopedic surgeons, while 44 (31.0%) physicians were from other backgrounds. Orthopedic surgeons described the tumor size more frequently than non-orthopedic physicians, indicating that orthopedic surgeons pay closer attention to the tumor size during the examination of soft tissue tumors. Although the result is unsatisfactory, it indicated that the guidelines were more commonly followed by orthopedic surgeons.

In Japan, some patients with malignant soft tissue tumors first visit the general clinic and are then referred to a cancer-specialized institution by the regional hospital. Alternatively, other patients are directly referred to a cancer-specialized institution by their local hospital. Ideally, both physicians working at clinics and regional general hospitals must be familiar with the guidelines for assessing soft tissue tumors. In this study, there was no significant difference between these two groups, suggesting that the commitment to accurately diagnose malignant soft tissue tumors is not different between these two groups.

Malignant soft tissue tumors are very rare. Fossum et al.^15^ reported that general physicians have low exposure to malignant soft tissue tumor cases, i.e., only 2.2 such cases over their entire careers, and they are also not familiar with the clinical guidelines for soft tissue sarcoma. Insufficient patient referral documents may cause delayed diagnosis, and recently, delayed diagnosis is reported to be the primary cause of sarcoma litigation^16^. Orthopedic oncologists play an important role in enhancing the awareness of general physicians, practicing the guidelines of soft tissue tumor management, and minimizing the misdiagnosis and unplanned resection of malignant soft tissue tumors.

This study had some limitations. First, this was a localized single-center small study in Japan and its results cannot be generalized to other countries. However, the referral trend of the patients with malignant soft tissue tumors may be important to enhance the awareness of general physicians and to prevent doctor-associated diagnostic delay. Second, the number of patients in this study was small. Third, the availability of information in a patient referral document could be assessed, but the presence or absence of knowledge regarding malignant soft tissue tumors among general physicians could not be correctly evaluated. Finally, clinical data on tumor size and depth were collected from MRI findings instead of physical findings. Although clinical findings are first assessed for diagnosing malignant soft tissue tumors, they are subjective, i.e., clinical information on the tumor size and depth could be dependent on each physician and it could be sometimes inaccurate.

## Conclusion

The referral documents of patients with typical findings of malignant soft tissue tumors were reviewed to investigate the application of the JOA clinical practice guidelines on the management of soft tissue tumors. The description rate of tumor size was approximately half, but that of tumor depth was even lower. The awareness of tumor size in diagnosing malignant soft tissue tumors has gradually improved. Orthopedic surgeons especially paid closer attention to the tumor size than non-orthopedic physicians, suggesting that the guidelines might be more prevalent among orthopedic surgeons. There is still a need to enhance the awareness of malignant soft tissue tumors among general physicians.

## Supplementary Information

Supplementary Information.

## Data Availability

The datasets used and/or analysed during the current study available from the corresponding author on reasonable request.
